# Impact of Ethanol and Saccharin on Fecal Microbiome in Pregnant and Non-Pregnant Mice

**DOI:** 10.4172/2376-127X.1000193

**Published:** 2015-10-01

**Authors:** Matthew T Labrecque, D’eldra Malone, Katharine E Caldwell, Andrea M Allan

**Affiliations:** Department of Neurosciences, University of New Mexico Health Sciences Center, Albuquerque, NM 87131, USA

**Keywords:** Ethanol, Obesity, Immunomodulation, Microbiomes

## Abstract

Research identifying connections between the gastrointestinal flora and human health has developed at a rapid pace. Several studies link the gut microbiome to a variety of biological functions beyond the gastrointestinal tract. Changes in our diets, including the consumption of artificial sweeteners, have profound effects on the composition of the gut microbiome and can, in turn, affect brain function, glucose tolerance, and inflammation.

Sweeteners are often used to encourage consumption of agents such as ethanol and nicotine in laboratory studies using rodents. Studies aiming to examine the effects of agents like ethanol on the developing nervous system administer these agents during pregnancy. To date, there have been no studies exploring the impact of the combination of dietary ethanol and saccharin during pregnancy on the gut microbiome in either humans or laboratory animal models.

In the study presented, we evaluated the impact of ethanol in either water or saccharin on the fecal microbiome in pregnant and non-pregnant mice using a qPCR approach. We found that the combination of ethanol and saccharin produced different effects than ethanol in water, depending on pregnancy status. Levels of Clostridium were reduced in ethanol-saccharin but not ethanol-water drinking mice, even though the total levels of ethanol consumed were the same for the two groups. Eubacteria were increased in the pregnant, but decreased in the non-pregnant, ethanol-saccharin drinking group. These treatment and pregnancy specific changes could impact the development of the offspring. In developing and quality checking our primer sets for these studies we identified several problems within previous research in the field. The technical drawbacks in previous studies, as well as our own study, are discussed. Despite some progress in the ability to study the gut microbiome, more advances and standardization of practices should be established to improve the reliability and validity of microbiome research.

## Introduction

Gut microbiomes have long been implicated in digestive health, though new research suggests that they play a role in a great many biological functions beyond the gastrointestinal tract. Aberrant gut microbiota composition may play a role in obesity and metabolic syndrome, insulin resistance, cardiovascular risk, and immunomodulation [1–4]. Influences of the environment on gut microbiomes have demonstrated that changes in our environmental location and our diets can have profound effects on the composition of the gut microbiome.

Current research has indicated the importance of the connection between the presence of the gut microbiome and the brain in everything from regulation of stress to development of neurologic disorders such as autism spectrum disorder [5]. Inflammation linked to gut dysbiosis and subsequent increased intestinal permeability has been linked to other neurologic, autoimmune disorders such as multiple sclerosis [6]. Alterations in gut permeability have recently been linked to the severity of alcohol dependence. Leclercq et al. [7], demonstrated that in subjects with alcohol dependence, an increase in intestinal permeability correlated with both dysbiosis and an increased severity of behavioral symptoms [7]. Taken together, these findings indicate that the gut microbiome is not only important to the maintenance of the impermeability of the gut mucosa, but also that a breach of this barrier may be important in the development of neuropsychological symptoms.

The gut microbiota is highly responsive to its environment and can be altered by host diet [8]. As was demonstrated by Caesar et al. [9] consumption of artificial sweeteners was demonstrated to cause functional alteration in the gut microbiomes of mice to such a degree that metabolic abnormalities were induced. Suez et al. [10] found that saccharin, a non-caloric artificial sweetener, had an adverse effect on gut microbiota configuration and induced glucose intolerance. Saccharin is commonly used to sweeten drinking treatments in laboratory mice that the animals may otherwise find undesirable. Ethanol consumption has also been shown to have a deleterious influence on the rodent gut microbiota [11]. Gohir et al. [12] recently concluded that pregnancy status impacts dietary effects on intestinal flora in mice. In our exposure paradigm, C57BL/6J female mice are given either 0.066% (w/v) saccharin solution alone (control) or 10% (w/v) ethanol in 0.066% saccharin solution (treatment) throughout breeding and pregnancy [13]. The mice are permitted ad lib food throughout and tap water was provided during the hours that ethanol is not presented. Given the current literature on the effects of saccharin and ethanol on gut microbiota and the potential health effects, in the present study we sought to determine the outcome of our exposure paradigm on the dam gut microbiota by measuring the fecal microbiome configuration.

Despite this rapid progress in understanding the role of the gut microbiome in health and disease, limitations exist in the ability to study such connections in collection and data analysis [5]. Studies of artificially reared mice showed significant variations in gut microbiome composition when compared to maternally reared mice [14]. Further, concern has been raised regarding the poor correlation that exists between the fecal and cecal microbiota profiles, indicating the difficulty in obtaining an accurate sample of laboratory animal gut microbiomes [15]. A review of the literature uncovered two primary methods for analyzing fecal microbiome composition utilizing the 16S RNA gene (16S rDNA), Next Generation Sequencing (NGS) and Quantitative Real-Time PCR (qPCR). NGS has the benefit of being powerful enough to accurately and quantifiably assess a large range of bacterial species within a sample. NGS can be cost prohibitive though and qPCR offers a less expensive alternative. The majority of the microbiome studies have used the qPCR method. In order to assess the sensitivity and validity of this approach, we utilized the qRT-PCR method. However, qPCR presents other challenges such as primer design and results analysis and interpretation. In this study, we will also examine some of the obstacles associated with 16S rDNA determination using qPCR.

## Methods

### Mice

C57BL/6J male and female mice were received from Jackson Labs at approximately 2 months of age. Both males and females were housed in reverse light cycle rooms with darkness occurring between 8:00 AM and 8:00 PM. Female mice were provided one of the four following solutions in tubes: 10% (w/v) ethanol and 0.066% (w/v) saccharin in water, 10% (w/v) ethanol in water, 0.066% (w/v) saccharin in water, and water. Tubes were placed on cages from 10:00 AM to 2:00 PM, daily. Thus, mice drank the ethanol saccharin or ethanol water solutions for only four hours per day. Those females consuming alcoholic solutions were incrementally increased from 0% ethanol to 10% ethanol. Female mice had been drinking solutions for 2 weeks before they were mated with C57 males. Three days prior to mating, soiled male bedding was added to a clean cage to which the female was transferred. When mating the mice, females were placed in the male’s cage at 2:00 PM and returned to their home cages at 8:00 AM the following day.

### Fecal collection

Females were weighed 1 day, 7 days, 9 days, 11 days and 15 days following breeding to gauge pregnancy status. Female fecal pellets were collected between 11 and 15 days after breeding. Female cages were changed and supplied with new, clean bedding 24 hours before fecal collection. Fecal samples were collected by sifting dirty bedding through a clean strainer. Pellets were collected individually out of the siftings and any attached bedding was removed. Bare fecal pellets were stored at −80ºC until used for DNA extraction.

### Bacterial DNA extraction

Bacterial DNA was extracted from 50 mg of weighed fecal pellets using the QIAmp DNA Stool Mini Kit (cat #: 51504; Qiagen, Valencia, CA). Modifications were made to the standard protocol provided in the kit handbook. *First*, the homogenate was incubated for 10 minutes at 70ºC and for 5 minutes at 95ºC (step 3). *Second*, the InhibitEX tablet was ground to a powder and added to the sample (step 5). *Third*, the sample was treated with PureLink RNase A (cat #: 12091-021; Life Technologies, Grand Island, NY) and allowed 2 minutes to incubate at room temperature (step 10). *Fourth*, high liquid volumes of supernatant were divided into volumes of no more than 500 mL (step 10). *Fifth*, 500 μL of AL Buffer and 500 μL Ethanol were added to the sample (step 11, step 13). *Sixth*, the DNA sample was eluted in 50 μL of AE buffer (step 18). DNA integrity was tested using a NanoDrop 1000 Spectrophotometer (Thermo Scientific, Waltham, MA) and quantified using the Qubit® dsDNA HS Assay Kit (cat #: Q32851; Life Technologies) on a Qubit® 2.0 Fluorometer.

### Quantitative real-time PCR

qPCR primers specific to the 16S ribosomal RNA gene of several bacterial groups were designed using the NCBI Primer-BLAST online software (Table 1) [16] with sequences obtained from NCBI GenBank. Primers were designed to include a maximum number of species within a pre-set group while excluding species from separate groups. Primer efficiencies were determined and primer concentrations were adjusted to provide efficiencies of 90 – 110% where possible. qPCR dissociation curves and agarose gel electrophoresis were utilized to determine primer specificity. qPCR was conducted on a LightCycler 96 instrument (Roche Diagnostics, Indianapolis, IN) with Roche FastStart Essential DNA Green Master (cat #: 06402712001) under standard profile conditions. Results were analyzed using the relative quantification method with an “All Eubacteria” primer set general to all eubacteria species as the endogenous control. Where primer efficiencies of targets differed from the endogenous control by more than 5%, Cq values were adjusted using the formula: Adjusted Cq = LOG (Amplification Factor^Initial Cq^), 2). Statistical significance (p<0.05) between the water and ethanol and the saccharin and saccharin/ethanol exposure groups was determined by Student’s *t*-test using GraphPad Prism 6 software (GraphPad Software, Inc., La Jolla, CA).

## Results

Average daily consumption during the 4 hour time period of ethanol and saccharin in water (1.1 mL +/− 0.05) and ethanol in water (1.0 mL +/− 0.07) was not different between the groups. The weights of pregnant mice in both drinking conditions were similar (32.75 g +/− 1.86 and 30.6g +/− 1.6 for saccharin-ethanol and water-ethanol, respectively) at the two week time point. Equal concentrations of ethanol consumed in saccharin water solutions produces the same blood alcohol levels as those consumed in water alone [17]. Mice in the two groups had the same average starting weight prior to pregnancy (19.0 g) indicating that the ethanol solutions, whether in water or in saccharin, did not alter weight gained during pregnancy.

**Figure 1:**
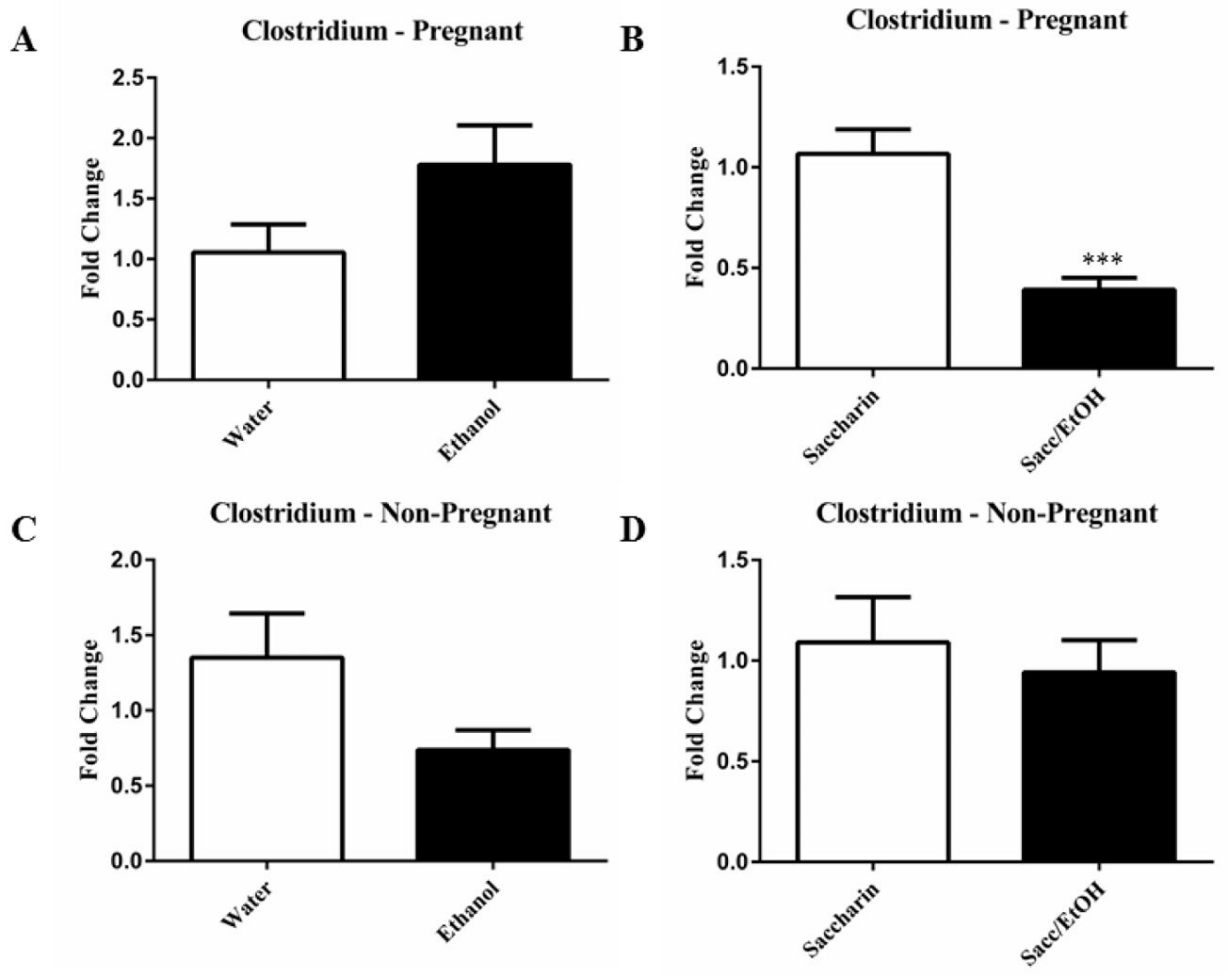
Drinking ethanol and saccharin together reduced levels of Clostridium in pregnant mice Ethanol in water solutions increased Clostridium levels in pregnant animals (1A) and decreased in non-pregnant animals (1C) but this did not reach significance. Ethanol and saccharin in water (1B) significantly decreased Clostridium levels in pregnant animals. No change was found in non-pregnant ethanol and saccharin in water drinking animals (1D). Data are expressed as mean fold change ± SEM, n=9–10 mice. Ethanol conditions are presented in the filled columns and controls (water or saccharin alone) are in the unfilled columns. *** t (17) =4.85, p=0.0001, n=9–10 (1A).

**Figure 2:**
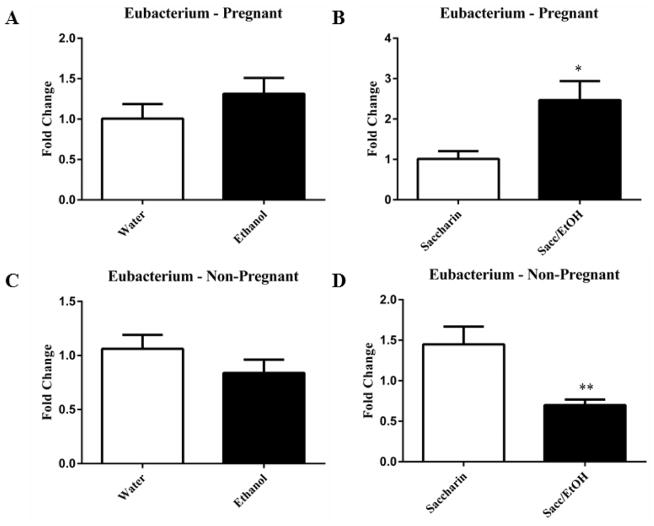
Drinking ethanol and saccharin together elevated levels of Eubacterium in pregnant mice but decreased it in non-pregnant mice Ethanol in water solutions did not affect the levels of Eubacterium in either pregnant (2A) or non-pregnant animals (2C). Ethanol and saccharin in water solutions significantly increased Eubacterium levels in pregnant animals (2B) and significantly decreased Eubacterium levels in non-pregnant (2D) animals. Data are expressed as mean fold change ± SEM, n=8–10 mice, t (13) =2.87, *p=0.01(2B) and t (14) =3.23, **p=0.006 (2D). Ethanol drinking conditions are presented in the filled columns and control (water or saccharin alone) are in the unfilled columns.

**Figure 3:**
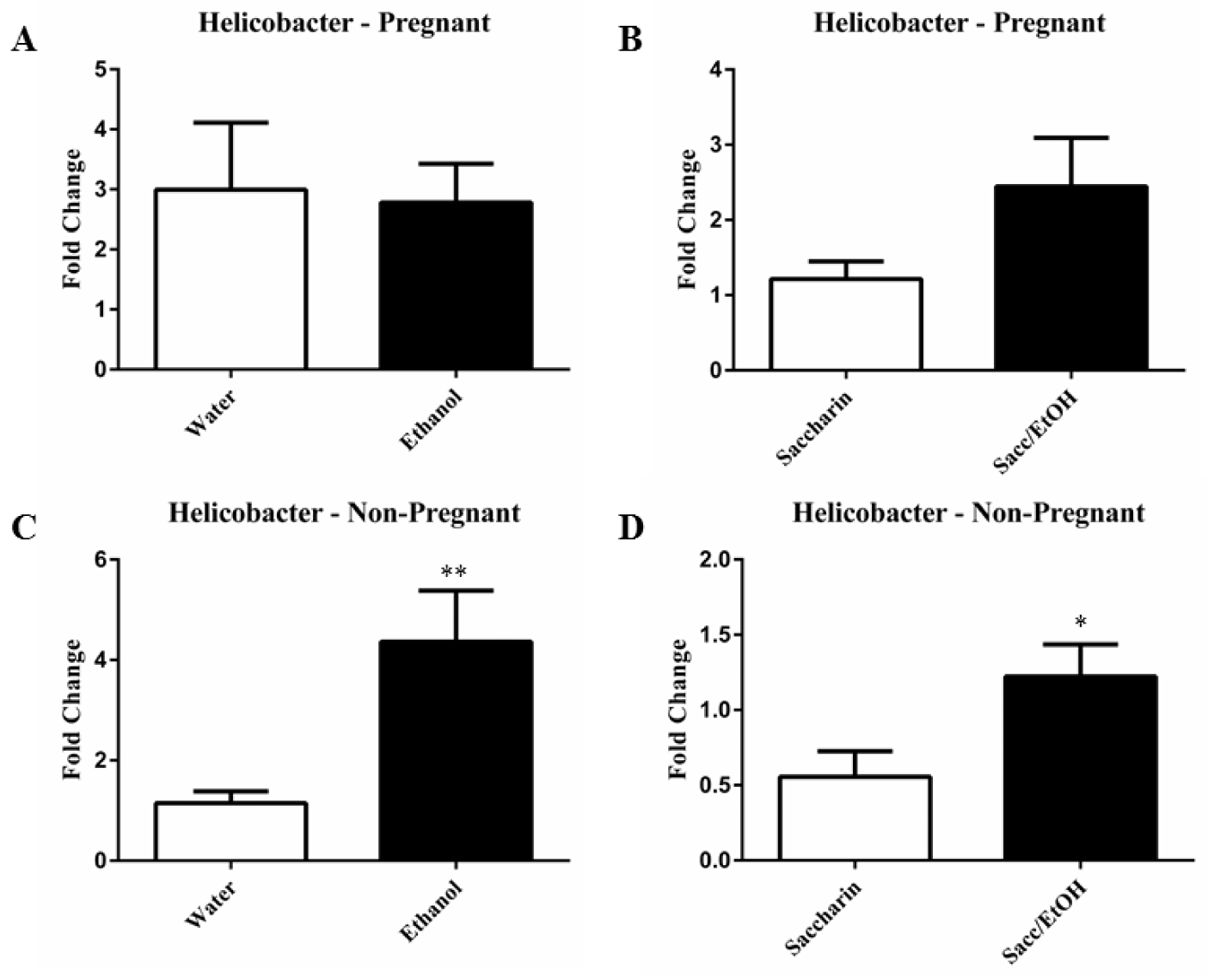
Helicobacter levels are increased in non-pregnant mice in response to ethanol consumption Ethanol in water solutions (3C) and ethanol and saccharin in water solutions (3D) significantly increased Helicobacter levels in non-pregnant animals. Ethanol and saccharin in water solutions increased Helicobacter levels in pregnant (3B) animals, but this did not reach significance. Data are expressed as mean fold change ± SEM, n=8–10 mice, t (16) = 2.76, **p=0.01 (3C) and t (15) =2.40, *p=0.03 (3D). Ethanol drinking conditions are presented in the filled columns and control (water or saccharin alone) are in the unfilled columns.

qPCR was utilized to examine the effect of ethanol (10% w/v) combined with either water or 0.066% (w/v) saccharin solution on pregnant and non-pregnant dam fecal microbiome content. Ethanol significantly decreased Clostridium levels in pregnant mice exposed to saccharin, p=0.0001 (Figure 1). Ethanol elevated Clostridium levels in pregnant mice drinking water and decreased levels in non-pregnant mice drinking water, however these measures did not reach significance. No change was found in the non-pregnant saccharin-drinking group. Ethanol significantly increased Eubacterium levels in pregnant saccharin mice and significantly decreased levels in non-pregnant saccharin drinking mice, p=0.01 and p=0.006, respectively (Figure 2). The pregnant water and non-pregnant saccharin animals followed these respective trends, but they did not reach significance. Ethanol exposure significantly increased Helicobacter levels in non-pregnant water and saccharin groups, p=0.01 and p=0.03, respectively (Figure 3). Helicobacter levels were also elevated in ethanol and saccharin in water-consuming pregnant mice but not to significance. No changes were observed in the pregnant ethanol in water group for helicobacter (Figure 3). Ethanol exposure decreased Bacillus levels in all test groups, particularly in the non-pregnant animals, however significance was not obtained (Supplementary Figure 1). Ethanol exposure did not yield observable differences in Bacteroides and Lactobacillus levels (Supplementary Figures 2 and 3). Ethanol exposure significantly decreased total eubacteria 16S rDNA levels in pregnant/water mice, p=0.03 (Supplementary Figure 4). 16S rDNA levels were elevated in ethanol exposed non-pregnant/water mice however they did not reach significance. No changes were found in the two other test groups.

## Discussion

It is well known that chronic consumption of significant amounts of alcohol leads to gastrointestinal mucosal damage, which, in turn, has been linked to changes in the gut microbiome environment [18–20]. While the majority of ethanol metabolism occurs within the liver and a portion within the gastrointestinal system, particularly in males, this metabolic process can generate reactive intermediaries, such as acetaldehyde, which can produce damage as well [21]. However, the impact of low-to-moderate levels of ethanol consumption in combination with artificial sweeteners on the fecal microbiome has not been evaluated. Further, although pregnancy has been shown to alter microbiome composition [12], the impact of pregnancy status has largely been ignored. These findings presented here demonstrate distinct interactions between pregnancy status and ethanol in water and ethanol and saccharin in water exposures. The changes in response to ethanol consumption depended, in part, on the presence or absence of saccharin and on the pregnant status of the mouse at the time of fecal sampling. For example, Clostridium levels were significantly reduced in the pregnant mice exposed to ethanol and saccharin but no difference was found in non-pregnant ethanol and saccharin in water exposed mice compared to control. While Clostridium levels were normal in non-pregnant ethanol and saccharin drinking mice, they were reduced in non-pregnant ethanol in water drinking mice. Eubacterium was significantly elevated in the pregnant ethanol and saccharin drinking mice but significantly reduced in non-pregnant ethanol and saccharin drinking mice. Similarly, Helicobacter was elevated by ethanol in either water or saccharin but only in non-pregnant mice. Ethanol reduced Bacillus levels in both drinking conditions in pregnant and non-pregnant mice, although not significantly. However, both Bacteroides and Lactobacillus levels were largely unaffected by the ethanol exposures in all test groups. It was also determined that ethanol and saccharin in water altered bacterial levels differently than ethanol in water. As mentioned above, Eubacterium levels were significantly reduced in non-pregnant ethanol and saccharin in water drinking mice however they were normal in non-pregnant ethanol in water drinking mice. Just what impact these changes in the fecal microbiome portend on the gut microbiome of the offspring is yet to be determined, but do suggest that there are microbiome changes associated with low to moderate consumption of ethanol and are affected by the presence or absence of a sweetening agent like saccharin.

The cornerstone of conducting a fecal microbiome study using qPCR is the ability to develop functional primer sets. This falls into two categories: basic understanding of qPCR methodology and target specificity, which are not independent of one another. A survey of literature uncovered many instances where 16S rDNA qPCR was conducted outside of the standards of amplicon length, primer Tm, and Self Complimentary and Self 3′ Complimentary scores. Commonly cited Lactobacillus group primers were originally designed by Walter et al. [22] and Heilig et al. [23] for end-point PCR. Those authors verified functionality of the primers for end-point PCR; however, it cannot be assumed that they are effective for qPCR reactions, especially given the 341 bp amplicon is well above the normal upper limit of 150 bp for qPCR. Amplicon size can dramatically impact qPCR reaction efficiency and target bias and make it more difficult to resolve multiple products on a dissociation curve. Should previously cited primers be used, it is vital that researchers verify functionality and reaction efficiency under current conditions. If primer efficiency differences have not been resolved, then there is a propagation of error. In the current study, the Enterobacteriaceae primers that were designed failed to meet qPCR efficiency requirements. Even with a mathematical efficiency correction, the results were far too variable to draw any conclusions (Supplementary Figure 5). Primer set design may prejudice results and confirmation using alternative primer sets should be considered.

The 16S ribosomal RNA gene contains a large degree of homology between bacterial species. This poses a significant problem when attempting to design primers. It can be a great challenge finding a primer set that meets the qPCR standards mentioned above and also targets only the species of interest. It is imperative for researchers to consult the most up-to-date genomic sequence databases. Primer sets identified from literature had been described as specific to certain bacterial species and were, in fact, perfectly matched to a broad range of other species, often overlapping with intended targets of other primer sets. Another difficulty is reconciling taxonomy with gene sequence. There were significant differences in 16S ribosomal RNA gene sequence between species within the same genera in several cases. Often, two or three sub-groups were represented by common sequences. Regions of sequence that were representative of the group as a whole and were still viable options for primers had to be determined. This could be achieved in some cases, but in others, some species would be mismatched in primer sequence. This also resulted in a small optimal range for amplicon size and sequence for primer sets. In the present study, a sample list of included genus/species for each primer set was generated as was a sample list of cross-detected genus/species (Table 1). An example of sequence variability for the Clostridium group is included in Supplementary Figure 6. If greater specificity is desired, then a nested primer approach is recommended [24]. The inclusion or exclusion of species will impact how results are interpreted.

Further complicating microbiome analysis with qPCR is that most bacterial species have multiple copies of the 16S ribosomal RNA gene, ranging from 1–15, spread throughout their genome [25]. Even within an individual species, environment can have an impact on the gene copy number. 16S rDNA qPCR is not a measure of microbiota cellular composition but instead a measure 16S rRNA gene copy number and results should be interpreted as such [24,26]. Calculating a percentage of total bacteria based solely on the ΔCq of a specific target fails to take this into account. The ΔCq provides a measure of the reaction specific to the primer targets and characteristics of that reaction. A direct comparison between two targets based on their respective ΔCq values cannot be made without normalizing to a standard curve [24,26,27] or estimating gene copy number based on genome size [28]. If absolute 16S rDNA gene copy values have not been established, then the only reasonable way to express results is as a fold change within a target normalized to the experimental control, as would be done with other relative quantification qPCR assays such as mRNA expression analysis. While this limits our understanding of microbiome composition, it allows for proper detection of changes as a result of experimental exposure.

The current study illustrates another potential problem with using 16S rDNA as a microbiome measure. Relative quantification analysis with qPCR can only be conducted with an endogenous control that is unaffected by the experimental conditions. It has been determined that the combination of pregnancy and ethanol exposure may lower the copy number of 16S rDNA in the total microbiome versus pregnancy and water. The results are based on a comparative measure of the raw Cq values with three separate qPCR runs per sample averaged (Supplementary Figure 2). Although only an estimation, this finding could confound the null results within the pregnant water/ethanol portion of this study. No significant differences were found in total microbiome raw Cq values for the other experimental groups.

In summary, the present study found that bacterial groups responded to ethanol solutions depending upon both the presence of an artificial sweetener and to pregnancy status. These changes in the fecal microbiome may have subtle effects on the health of the offspring which should be considered in future studies. While there are drawbacks to using 16S rDNA qPCR analysis due to inherent limitations, it remains a viable tool and represents an inexpensive alternative to next generation sequencing. More advances and standardization of practices are needed to improve the reliability and validity of microbiome research.

## Supplementary Material

Supplemental figures

## Figures and Tables

**Table 1:** Quantitative real-time PCR primer information.

All Eubacteria							
	Sequence (5′->3′)	Length	Tm	Gene Copy #	Primer Efficiency %	Amp Factor	Primer Conc. (nM)
**Forward primer**	ACTCCTACGGGAGGCAGC	18	60.76	2–12	100.14	2	500
**Reverse primer**	ATTACCGCGGCTGCTGG	17	60.18				
**Product:**	~ 172–198						
**Included:**	Bacillus, Bacteroides, Candidatus, Clostridia, Enterobacter, Eschericia, Eubacterium, Helicobacter, Lactobacillus, Salmonella, Yersinia						
**Bacillus**							
	**Sequence (5′->3′)**	**Length**	**Tm**	**Gene Copy #**	**Primer Efficiency %**	**Amp Factor**	**Primer Conc. (nM)**
**Forward primer**	CCACACTGGGACTGAGACAC	20	59.97	12	100.98	2.01	50
**Reverse primer**	CCGTGGCTTTCTGGTTAGGT	20	59.96				
**Product:**	196–197 bp						
**Included:**	Bacillus, Oceanobacillus, *Enterococcus, Listeria, Staphylococcus*						
**Bacteroides**							
	**Sequence (5′->3′)**	**Length**	**Tm**	**Gene Copy #**	**Primer Efficiency %**	**Amp Factor**	**Primer Conc. (nM)**
**Forward primer**	AGGAAGGTCCCCCACATTG	19	58.91	6	111.73	2.12	100
**Reverse primer**	CGCTACTTGGCTGGTTCAG	19	58.54				
**Product:**	105 bp						
**Included:**	Bacteroides, Prevotella, *Odoribacter, Parabacteroides, Tannerella*						
**Clostridium**							
	**Sequence (5′->3′)**	**Length**	**Tm**	**Gene Copy #**	**Primer Efficiency %**	**Amp Factor**	**Primer Conc. (nM)**
**Forward primer**	GGGAGTACGGTCGCAAGATT	20	59.82	12	95.87	1.96	250
**Reverse primer**	ATGCACCACCTGTCTTCCTG	20	59.96				
**Product:**	169–176 bp						
**Included:**	Clostridium						
**Enterobacteriaceae**							
	**Sequence (5′->3′)**	**Length**	**Tm**	**Gene Copy #**	**Primer Efficiency %**	**Amp Factor**	**Primer Conc. (nM)**
**Forward primer**	TATCCTTTGTTGCCAGCGGT	20	59.96	7	241.78	3.42	100
**Reverse primer**	CGCTTCTCTTTGTATGCGCC	20	59.97				
**Product:**	145 bp						
**Included:**	Enterobacter, Escherichia, Salmonella, Shigella						
**Eubacterium**							
	**Sequence (5′->3′)**	**Length**	**Tm**	**Gene Copy #**	**Primer Efficiency %**	**Amp Factor**	**Primer Conc. (nM)**
**Forward primer**	GGGGAGTACGTTCGCAAGAA	20	60.04	5	105.09	2.05	250
**Reverse primer**	GCTCCGAAGAGAAGGTACGG	20	59.9				
**Product:**	152						
**Included:**	Eubacterium rectale						
**Helicobacter**							
	**Sequence (5′->3′)**	**Length**	**Tm**	**Gene Copy #**	**Primer Efficiency %**	**Amp Factor**	**Primer Conc. (nM)**
**Forward primer**	CAAGCCTGAAGCAGCAACG	19	60.08	2	129.36	2.29	500
**Reverse primer**	CGCCCAGTGATTCCGAGTAA	20	59.83				
**Product:**	165 bp						
**Included:**	Helicobacter bizzozeronii, cetorum, felis, *mustelae; Wolinella*						
**Non-Specific:**	Helicobacter cinaedi, hepaticus, pylori; Campylobacter						
**Lactobacillus**							
	**Sequence (5′->3′)**	**Length**	**Tm**	**Gene Copy #**	**Primer Efficiency %**	**Amp Factor**	**Primer Conc. (nM)**
**Forward primer**	GAGTACGACCGCAAGGTTGA	20	60.04	6	89.21	1.89	500
**Reverse primer**	CCCAACATCTCACGACACGA	20	60.04				
**Product:**	202 bp						
**Included:**	Enterococcus, Lactobacillus, Lactococcus, Leuconostoc						
**Cross-Detection:**	Bacillus, Eubacterium, Listeria, Staphylococcus, Streptococcus, Weissella, *Clostridium*						
