# Supplementary material for: Impact of Ethanol and Saccharin on Fecal Microbiome in Pregnant and Non-Pregnant Mice
Source: J Pregnancy Child Health. Author manuscript; Available in PMC 2016 Mar 15. (PMC4792281; doi:10.4172/2376-127X.1000193)
Supplement: Supplemental figures [file NIHMS737465-supplement-Supplemental_figures.pdf]

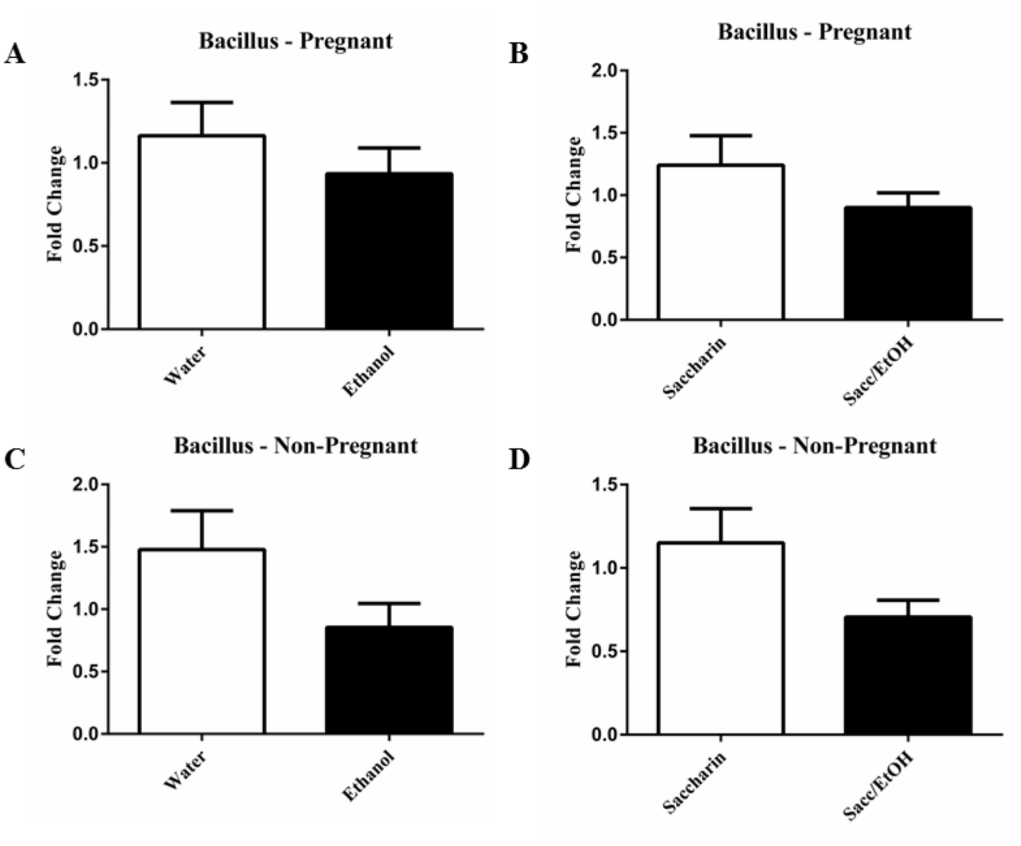

Supplementary Figure 1: Ethanol in either water or in a water and saccharin solution did not have a significant effect on Bacillus levels in either pregnant or non-pregnant mice. Data are expressed as mean fold change ± SEM, n=8-9 dams. Ethanol drinking conditions are presented in the filled columns and control (water or saccharin alone) are in the unfilled columns.

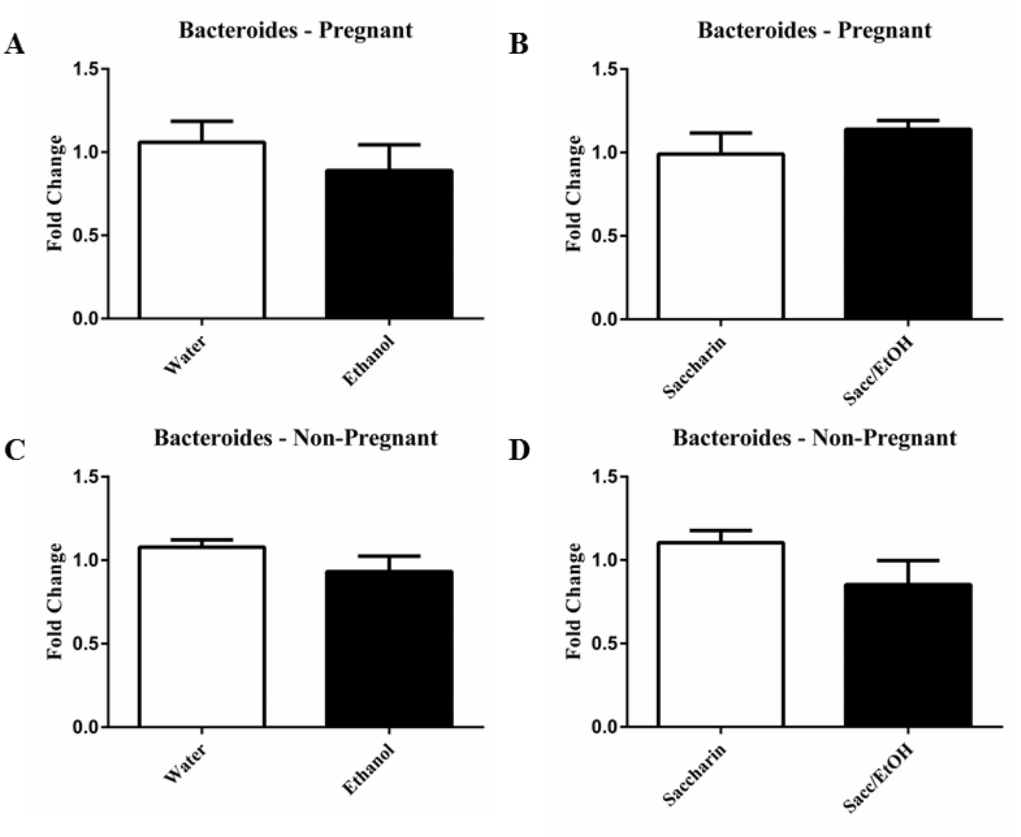

Supplementary Figure 2: Ethanol in either water or in a water and saccharin solution did not have a significant effect on Bacteroides levels in either pregnant or non-pregnant mice. Data are expressed as mean fold change ± SEM, n=8-10 dams. Ethanol drinking conditions are presented in the filled columns and control (water or saccharin alone) are in the unfilled columns.

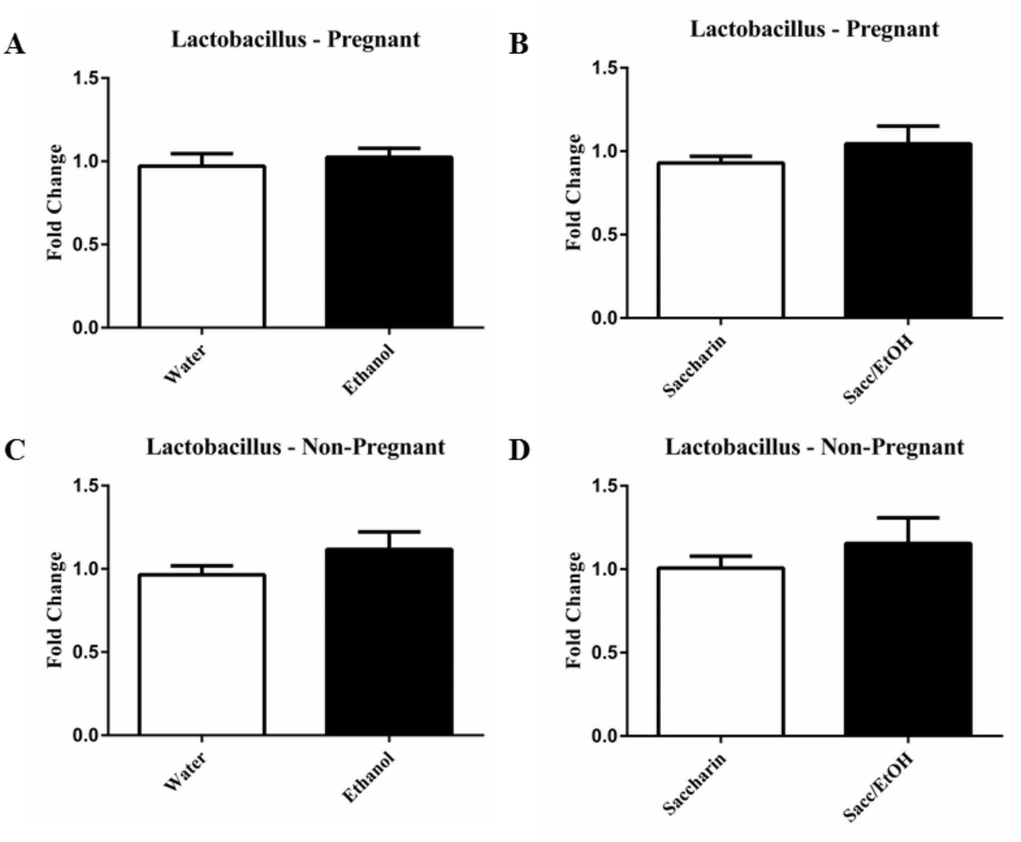

Supplementary Figure 3: Ethanol in either water or in a water and saccharin solution did not have a significant effect on Lactobacillus levels in either pregnant or non-pregnant mice. Data are expressed as mean fold change ± SEM, n=8-10 dams. Ethanol drinking conditions are presented in the filled columns and control (water or saccharin alone) are in the unfilled columns.

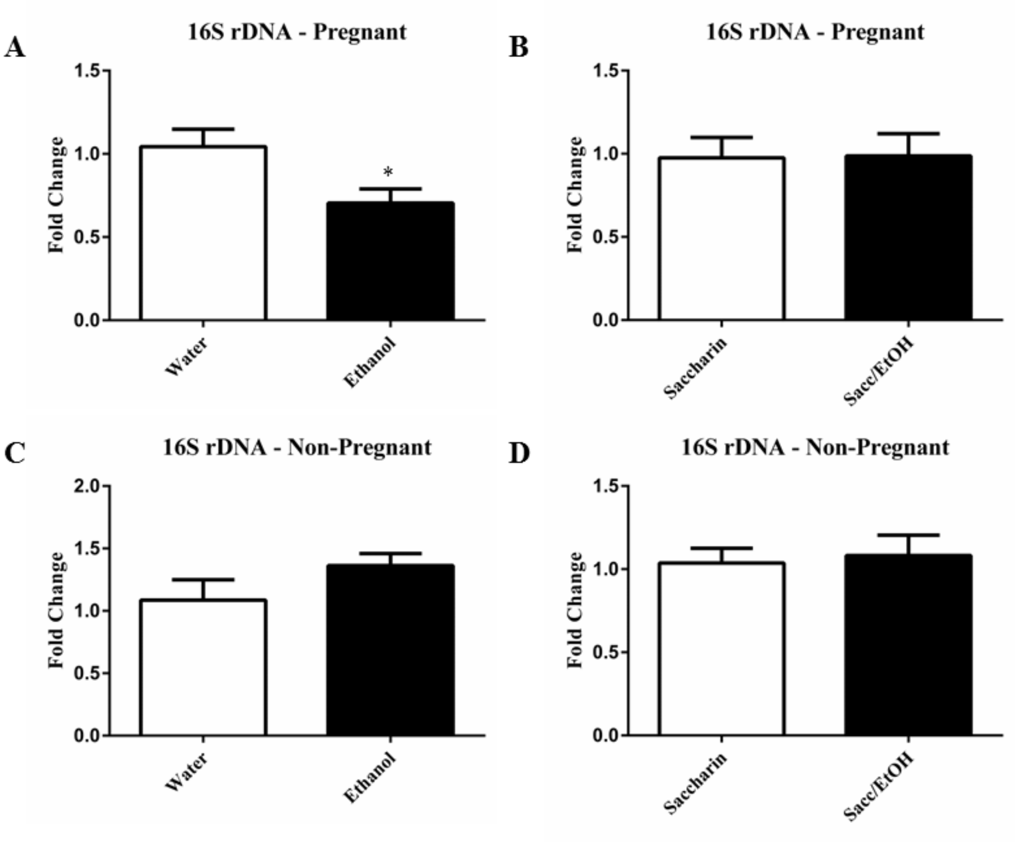

Supplementary Figure 4: Ethanol in water significantly decreased 16S rDNA copy number in total eubacteria of pregnant animals (suppl 2A). No changes were found in other groups. Data are expressed as mean fold change ± SEM, n=8-10 dams. Pregnant Water t (15) = 2.47, *p=0.03 (suppl 2A). Ethanol drinking conditions are presented in the filled columns and control (water or saccharin alone) are in the unfilled columns.

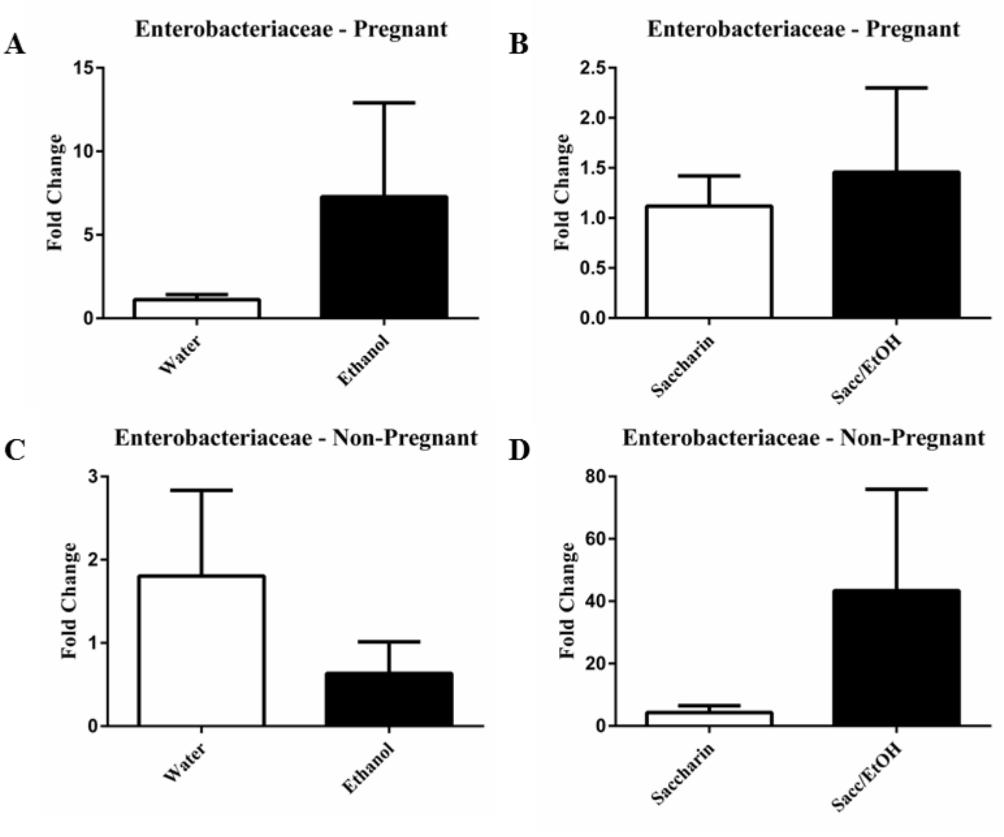

Supplementary Figure 5: Ethanol in either water or in a water and saccharin solution did not have a significant effect on Enterobacteriaceae levels in either pregnant or non-pregnant mice. Very high variability was observed because of the abnormally high reaction efficiency. Data are expressed as mean fold change ± SEM, n=8-10 dams. Ethanol drinking conditions are presented in the filled columns and control (water or saccharin alone) are in the unfilled columns.

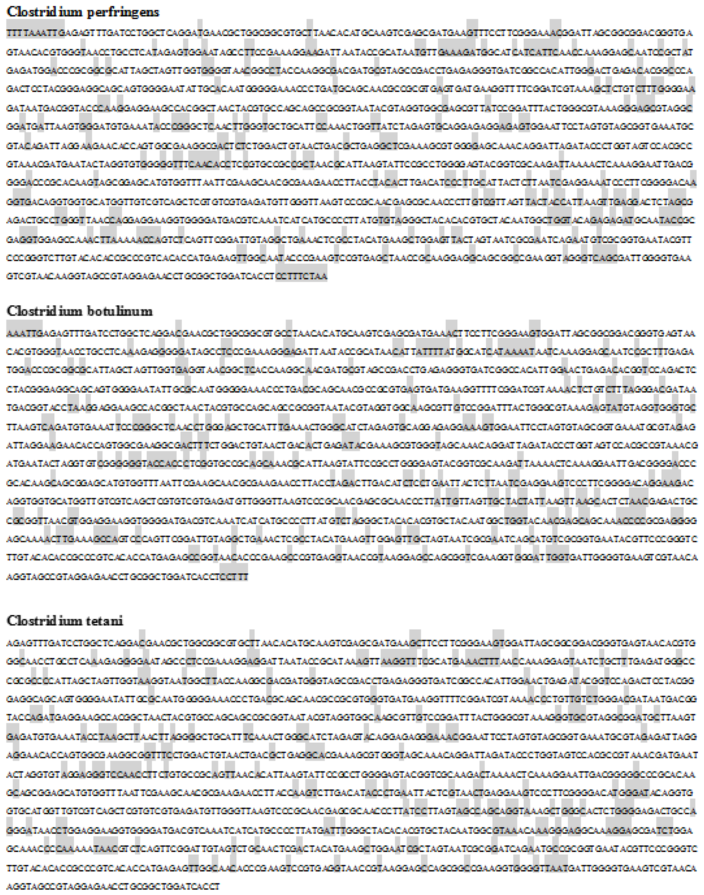

Supplementary Figure 6: 16S rDNA sequence for *Clostridium perfringens* str. 13 (NC_003366.1), *Clostridium botulinum* BKT015925 (NC_015425.1, *discontinued*), and *Clostridium tetani* 12124569 (NC_022777.1). Nucleotides not aligned to all three sequences are highlighted in gray.
